# BioAcoustica: a free and open repository and analysis platform for bioacoustics

**DOI:** 10.1093/database/bav054

**Published:** 2015-06-08

**Authors:** Edward Baker, Ben W. Price, S. D. Rycroft, Jon Hill, Vincent S. Smith

**Affiliations:** ^1^Department of Life Sciences, Natural History Museum, Cromwell Road, London SW7 5BD, UK and ^2^Environment Department, University of York, Heslington, York YO10 5DD, UK

## Abstract

We describe an online open repository and analysis platform, BioAcoustica (http://bio.acousti.ca), for recordings of wildlife sounds. Recordings can be annotated using a crowdsourced approach, allowing voice introductions and sections with extraneous noise to be removed from analyses. This system is based on the Scratchpads virtual research environment, the BioVeL portal and the Taverna workflow management tool, which allows for analysis of recordings using a grid computing service. At present the analyses include spectrograms, oscillograms and dominant frequency analysis. Further analyses can be integrated to meet the needs of specific researchers or projects. Researchers can upload and annotate their recordings to supplement traditional publication.

**Database URL:** http://bio.acousti.ca

## Introduction

Collections of recorded wildlife sounds have huge potential as a resource for systematics, e.g. (1–3); biogeography, e.g. (4–6) and automated identification, e.g. (7–9). Many libraries of recorded sound exist in institutional collections but these are often underused, often because knowledge of their existence is limited within the institution, let alone outside of it. In order to maximize the potential of these collections they need to be easily accessible, and linked with the broader infrastructures of biodiversity informatics. Where collections are available, they generally do not deal with recordings containing multiple taxa or allow for annotation of regions containing spoken metadata or extraneous noise, e.g. (10).

The aims of BioAcoustica are 4-fold: (i), to make recordings available to as large an audience as possible in both human and machine readable formats; (ii) to facilitate crowdsourced annotation of recordings; (iii) to integrate common acoustic analyses without the need for additional specialist programs and (iv) provide the metadata of recordings to interested communities outside of the field of bioacoustics, e.g. the recording of a species in the wild is an observation of that species at a particular time and place. Records of this type are aggregated by the Global Biodiversity Informatics Facility (GBIF: http://gbif.org) and can then be used in studies of global distribution of species.

### Technical overview

**Figure 1.**
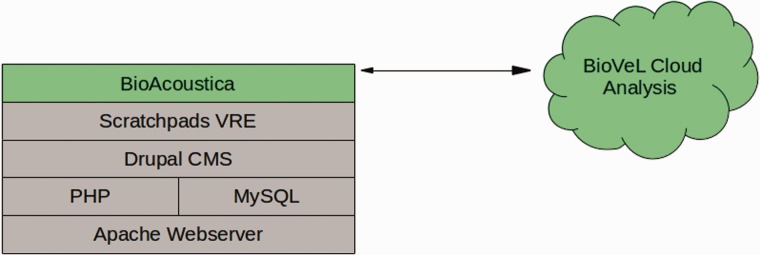
Infrastructure diagram of BioAcoustica, showing underlying server infrastructure (grey) and the systems.

BioAcoustica is a specialised instance of the Scratchpads (11) virtual research environment. Scratchpads provides a community website (a Scratchpad) for collaboratively managing biodiversity-related content, in this case audio files, locations, specimens, bibliographic references and the biological taxonomy used to manage them. The Scratchpad also manages user registration, commenting on content and the various methods of displaying content. Scratchpads are built on Drupal (http://drupal.org), an open source content management system. The BioAcoustica Scratchpad has been extended to allow for the annotation of audio files and for annotated sections of audio files to be analysed from within the Scratchpad by creating a custom content type (node type in Drupal) and additional code modules (using PHP and JavaScript) to handle the annotation and analysis of sounds. Analysis is performed by a cloud computation service. Figure 1 shows the infrastructure discussed in this article.

### Data model

Scratchpads provide node types for creating bibliographic, specimen/observation and location nodes, where a node is an individual item of content on a Scratchpad. The specimen/observation and location nodes follow the DarwinCore standard (12).

**Figure 2.**
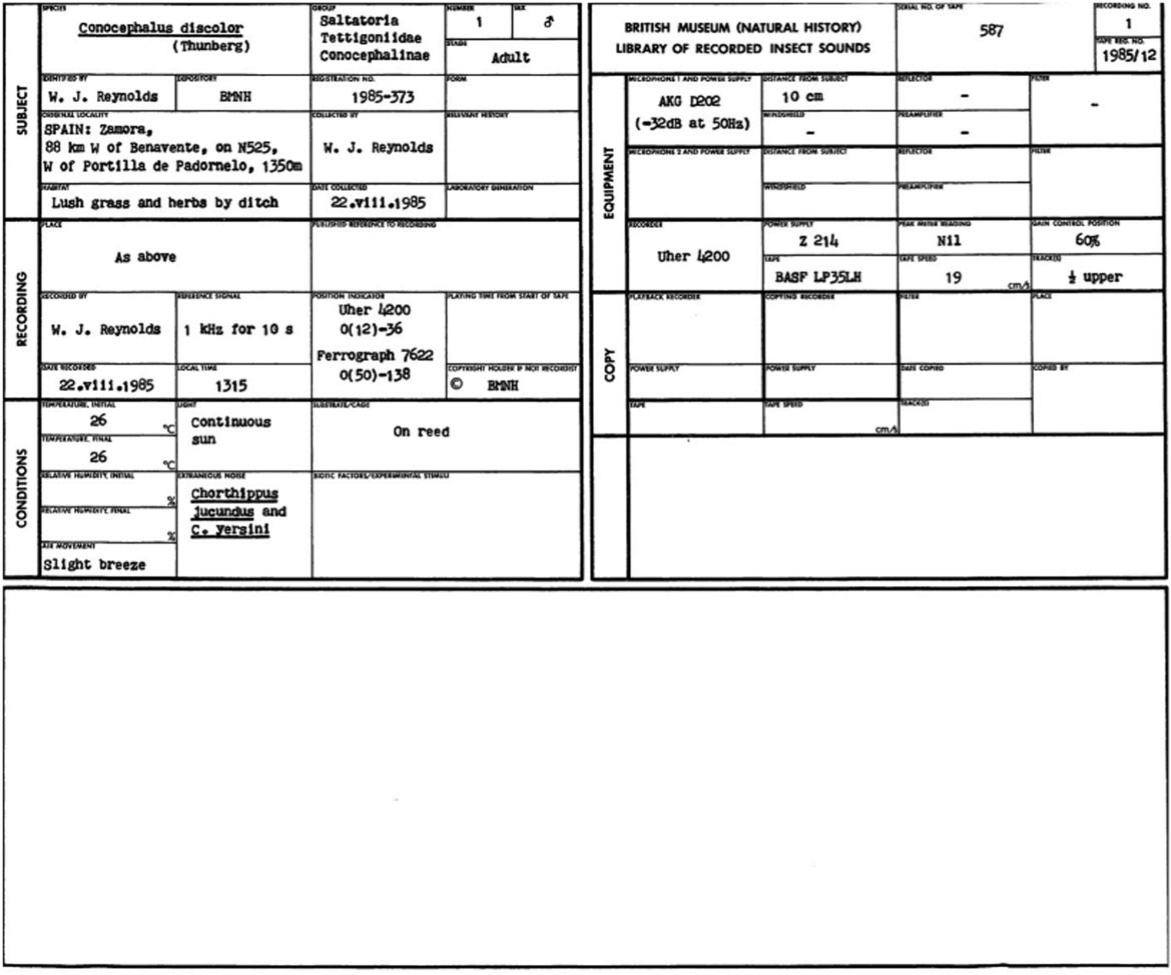
Example metadata record from the BMNH Acoustic Laboratory.

**Figure 3.**
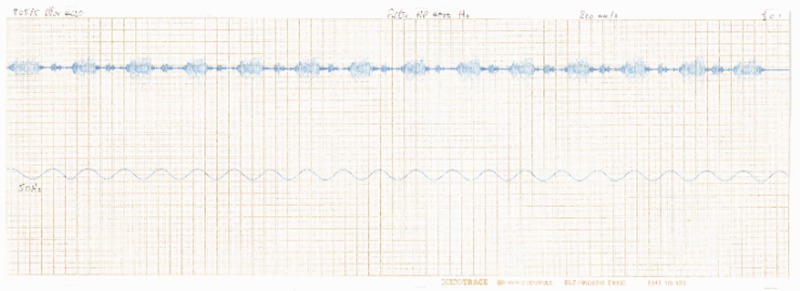
Scan of a waveform made in the BMNH Acoustic Laboratory. There exist a number of these wave traces where the original recording cannot be located.

The only new node type created in BioAcoustica is the ‘Recording’ content type which is used to store both the recorded file (in WAV format) and the associated metadata. Table 1 shows the metadata fields used. These fields are based on the original metadata sheets used by the BMNH Acoustic Laboratory (http://sounds.myspecies.info/node/11273; Figures 2 and 3).

**Table 1.** Metadata fields used in the BioAcoustica ‘Recording’ node type

Group	Field	Description	Example
	Title	The name used to identify the recording (typically includes original CD/tape number and species)	954 *Conocephalus discolor*
	Recording	The audio file	
	Project		Natural History Museum Sound Archive
**Source**	Original metadata image	A scan or photograph of the original metadata if it exists in printed or handwritten form	See Figure 1
	Original trace images	Scan(s) or photograph(s) of paper oscillograms relating to the recording	See Figure 2
	Original verbatim species	The species identification as recorded in the original metadata	*C. discolor*
	Original CD number	Used to associate digital record with physical collection	575
	Original CD track number	Used to associate digital record with physical collection	4
	Original tape number	Used to associate digital record with physical collection	575
	Copyright holder		Natural History Museum
	Licence		Creative Commons: Attribution
**Subject**	Species	Link to a taxon in the site’s biological classification	*C. discolor*
	Requested additional species	Used to suggest a name that is not currently in the site’s biological classification	
	Specimen	Link the recording to a preserved or observed specimen	BMNH–E–1427969
**Recording**	Location	Location the recording was made (may be different to where the specimen was collected)	BMNH Acoustic Laboratory
	Published reference	Link(s) to publications that make use of this recording	Price *et al.* (13)
	Recorded By	Who made the recording?	Ragge
	Date recorded	Date the recording was made	06.xi.1985
	Local time	Local time recording was made	13:00
	Reference signal	Frequency of any reference signal used	10 kHz
**Conditions**	Initial temperature	Temperature in Celsius at the start of the recording	23.5
	Final temperature	Temperature in Celsius at the end of the recording	24.0
	Initial relative humidity	Relative humidity (%) at start of recording	40
	Final relative humidity	Relative humidity (%) at end of recording	40
	Air movement		Light from fan 1 m away
	Light		60 W desk lamp
	Extraneous noise		Fan in background
	Substrate or cage		Mesh cage
	Biotic factors or experimental conditions		None
**Equipment: general**	Microphone and power supply		SennheiserMKH 405
**Equipment: recorder**	Recorder		Kudelski Nagra IV D
	Power supply		ATN2/QED750
	Peak meter reading		−20
	Gain control position		130
	Tape		BSF SP52
	Tape speed (cm/s)		38
	Tracks		Full

These are based on the fields used by the BMNH Acoustic Laboratory.

**Figure 4.**
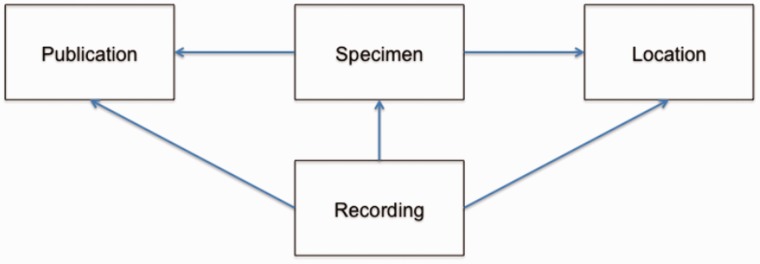
Data linkages in BioAcoustica. The creation of links is a unidirectional process, although back- and forward-links are presented to the end user.

BioAcoustica allows linkage of various nodes of different types to create a navigable resource covering recordings, specimens (whether living or later preserved), locations and publications. The linkages used in BioAcoustica are illustrated in Figure 4 and listed in Table 2 along with their rationales.

**Table 2.** Usage rationales for data linkages within the BioAcoustica website

From	To	Usage
Recording	Specimen	Multiple recordings may be made from a single specimen
Specimen	Location	Multiple specimens may be recorded in the same location; individuals may be recorded at the same population over a number of years (e.g. Chapman’s Pool for *Conocephalus discolor*)
Recording	Location	Was the recording made in the field, or elsewhere at a later time from a collected individual
Specimen	Publication	A publication may cite a specimen, or be used to provide further details about a specimen, e.g. using (14) to add GenBank references)
Recording	Publication	Allows a list of recordings used in a publication to be generated

**Figure 5.**
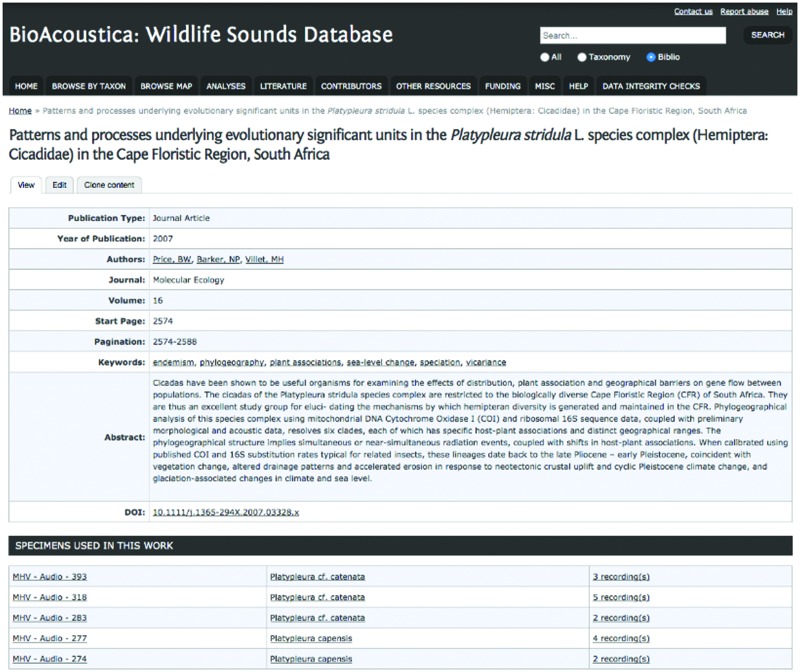
Publication page from BioAcoustica website showing links to specimens and recordings referenced in the article.

Linkages between different types of content are displayed to the user at the bottom of the relevant webpage. See Figure 5 for an example showing specimens used in a published research article.

### Waveform display

**Figure 6.**
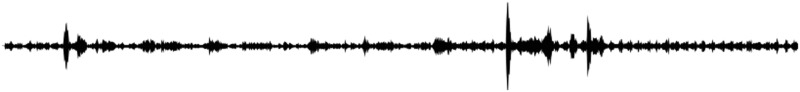
Waveform generated by the wavesurfer.js library (from: http://bio.acousti.ca/node/11778).

BioAcoustica stores recordings in waveform audio (preferred) or MP3 encoded format. Waveform images of audio files are displayed on recording pages, allowing the user to examine the overall shape of the waveform (Figure 6) and to facilitate precise annotations (see Annotation). These waveforms are generated in the user’s browser using the wavesurfer.js library (JavaScript). For longer audio files the waveform automatically scrolls across the screen as the file is played.

The waveform module for Drupal developed by Michael Mallet (see Code repositories) provides an integration of the wavesurfer.js code into the Drupal environment. The waveform module for Scratchpads developed for use in BioAcoustica is based on that by Michael Mallet with modifications by Baker and Rycroft to create a more comprehensive Drupal and Scratchpads integration, and to automatically scale the waveform to an appropriate size depending on the duration of the file.

In order to allow the waveform to be clearly visible while making annotations, and to allow accurate region selection, the waveform remains at the top of the page (at full width) as the user scrolls down. The horizontal (temporal) resolution of the waveform is limited to the size of the HTML canvas element that is used to draw it. The resolution is automatically set by BioAcoustica to allow maximum possible detail.

### Annotation

The BioAcoustica Scratchpad has been extended to allow for the annotation of audio files. Many recordings include a spoken introduction and/or contain periods of extraneous noise. These sections must be removed before performing analysis of that sound file. In addition, recordings may include the acoustic behaviour of one or more individuals of a single species, or of more than one species. The annotation function allows for different parts of the file to be labelled appropriately. These annotated sections may then be used in acoustic analyses.

The annotation functionality has been achieved through the modification of the commenting facility of Scratchpads that creates an additional type of comment that is used as an annotation. Annotations require the user to select a type of annotation (e.g. voice introduction, extraneous noise, call with extraneous noise, clear call), the start and end times of the annotated section in seconds and optionally a free-text description of the annotation.

When an annotation is saved the type of the annotation is checked, and if the audio section is specified as a call, then the section is queued for analysis.

**Figure 7.**
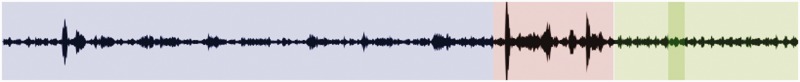
Annotated waveform generated using the Regions plugin for wavesurfer.js. Annotated regions are colour coded by the type of annotation (blue for voice introductions, red for extraneous noise and green for calls). From: http://bio.acousti.ca/node/11778.

Annotations’ waveforms are displayed using the Regions plugin for wavesurfer.js. The annotated regions are coloured based upon the type of the annotation (Figure 7). Regions are translucent to enable the visual display of overlapping annotations.

### Analysis

Analysis is performed through the Seewave package (15) for the R statistical language and environment (16). The Biodiversity Virtual e-Laboratory (BioVeL, Ref. 14) Portal is used to perform analyses. The BioVeL portal runs Taverna workflows (17) submitted by users or other services using the myGrid infrastructure (http://www.mygrid.org.uk/).

When an analysis is required, the Scratchpad sends a request to the BioVeL portal specifying the analysis to be performed, a link to the audio file to be analysed and any other required parameters (e.g. the start and end points of the analysis if the analysis is not of the entire file). The analysis is then queued by the BioVeL Portal and performed asynchronously from BioAcoustica. This method allows for multiple analyses to be submitted and performed without impacting on the performance of the Scratchpads server (18).

The Scratchpad periodically checks for completed analyses. When an analysis has completed the Scratchpad server downloads a zip file containing the analysis results from the BioVeL portal. The zip file is extracted and the results of the analysis are attached to the appropriate annotation. The analysis can be returned from the BioVeL portal, linked to the relevant annotation and updated in the user’s web browser without the user having to refresh their page due to the use of asynchronous web technologies.

This ‘analyse by default’ method is facilitated by using the external computational power provided by the BioVeL portal and provides for great efficiency: the analyses are completed quickly in the background as you annotate the sections you wish to study without competing for the resources of the Scratchpads servers.

### Types of analysis

**Figure 8.**
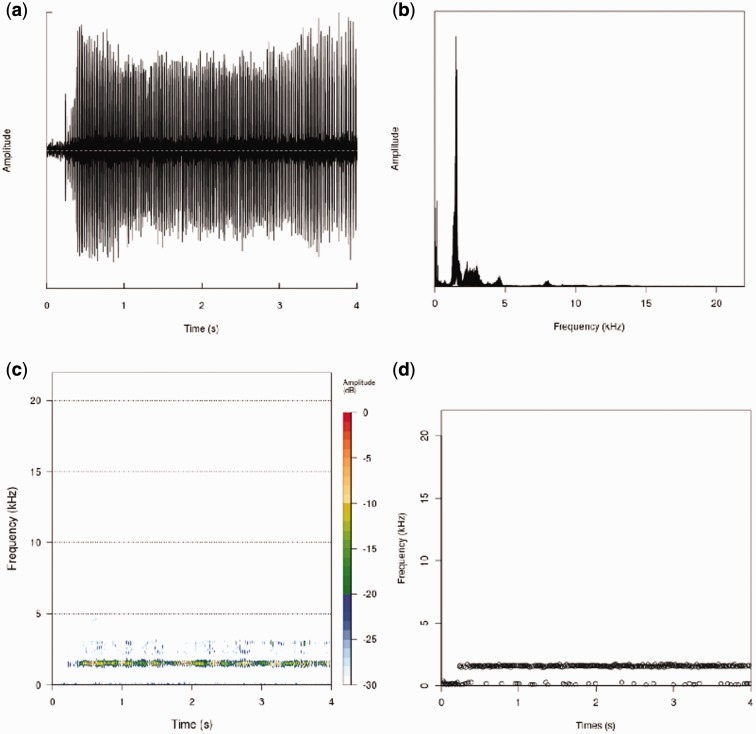
Analysis plots generated using the seewave package for the R statistical language running on the BioVeL portal (from http://bio.acousti.ca/comment/40#comment-40). (**a**) Oscillogram showing overall amplitude of wave against time, (**b**) plot of frequency amplitude against frequency, (**c**) spectrogram plot, heatmap of frequency against time, (**d**) dominant frequency (largest amplitude) against time.

The default analysis workflow of BioAcoustica generates the following widely used analyses: dominant frequency, spectrogram, frequency spectrum and acoustic complexity index (Figure 8).

Researchers may use the sound files contained in BioAcoustica to perform their own analyses on their local machines, or develop a Taverna workflow that can be run from the BioVeL portal. Creating a Taverna workflow has the advantage that it can be integrated into the BioAcoustica site if it is widely used.

**Figure 9.**
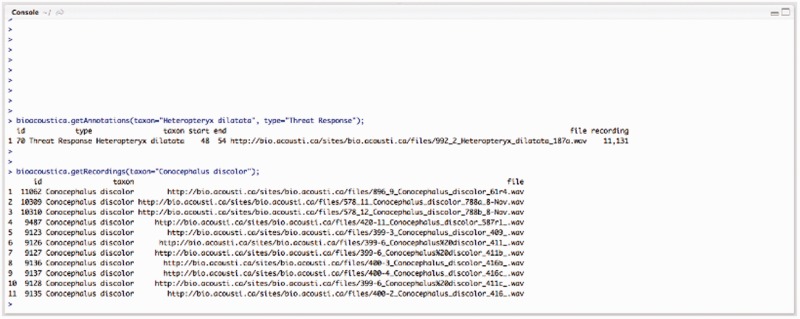
R console example of using bioacousticaR to query the BioAcoustica database of recordings and annotations.

The R package bioacousticaR, currently available from the Natural History Museum’s GitHub repository is being developed to allow querying of the BioAcoustica recordings and annotations from within an R environment (Figure 9).

### Viewing analyses

**Figure 10.**
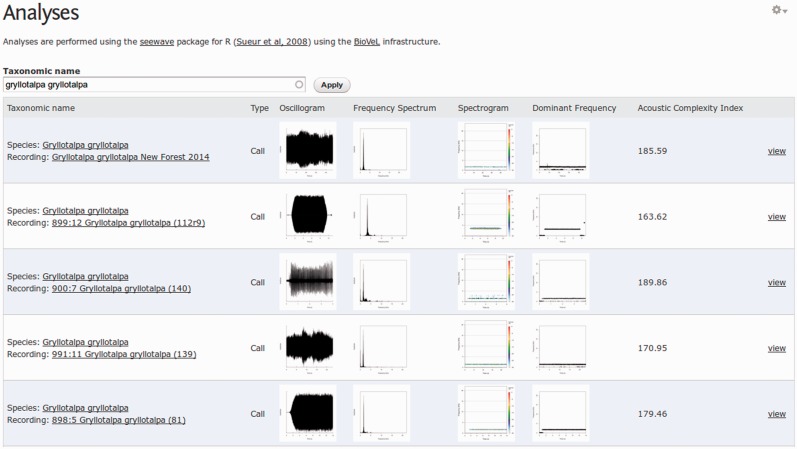
View of five analyses of different recordings of the European Mole Cricket *Gryllotalpa gryllotalpa*.

As well as being able to view analyses directly below the recordings on which they are based, we have also created specialized views of the analyses to enable comparisons to be easily made (Figure 10). A list of all analyses performed can be found at http://bio.acousti.ca/analyses. This list can be filtered by taxon.

### Taxonomy and nomenclature

The site uses a biological classification managed by the authors and limited in scope to taxa that have content within the database. External contributors can request the addition of taxa to this classification to accommodate their datasets. Mass imports of taxa are first checked against current taxonomies in the Encyclopedia of Life (EoL) through an automated script (19).

After the import of new datasets, scientific name strings are checked for synonyms and misspellings using the EoL database (20) using tools from the Supertree Toolkit (19). Each name in the sound database is checked by searching for that name via the EoL API. EoL returns information on possible synonyms within a JSON data object. For each name checked a status is assigned. These are: ‘green’: meaning the name is identical to the currently valid scientific name; ‘yellow’: meaning the name is a misspelling or synonym and the current valid scientific name is suggested; ‘amber’: the search return multiple results for the name and so is a homonym and required manual checking and ‘red’ meaning the name was not found.

Due to the nature of the EoL API manual checking of the results is required to see if scientific names on the BioAcoustica site need to be changed (e.g. issues may be raised by identical names that legitimately occur separately under the botanical and zoological codes of nomenclature). For this reason, name statuses are not shared with the public, but are available to site administrators and data providers.

### Data integrity

The database is backed up daily, and daily backups are kept for 1 week, a monthly backup is kept for 1 year and yearly backups are currently kept indefinitely. Files (including audio recordings) are backed up daily, and deleted files are kept for 6 weeks. These backups are to preserve the data integrity of the site, and are not intended to be a resource for end users beyond peace of mind.

The Natural History Museum is committed to ensuring continued support (both hardware resource and developers) for the Scratchpads project to ensure the longevity of this, and all other Scratchpad instances.

### Code repositories

The modifications to the Scratchpads code made by the BioAcoustica project are available in the Scratchpads repository. The code is separated into wildsound Drupal module, located at /sites/all/modules/custom/wildsound. All code listed in the repositories below is available under open licences, please see individual repositories for any restrictions as well as installation instructions.

- Scratchpads 2.0: https://git.scratchpads.eu/v/scratchpads-2.0.git
- R analysis scripts: https://github.com/NaturalHistory Museum/WildSoundDB-Scripts
- bioacousticaR: https://github.com/NaturalHistory Museum/bioacousticaR
- Issue Tracker: http://support.scratchpads.eu/projects/wildsound
- STK: https://code.launchpad.net/∼stk-developers/supertree-toolkit/stk
- Code from projects used:
- wavesurfer.js: https://github.com/katspaugh/wavesurfer.js
- Michael Mallet’s wavesurfer module: https://github.com/MichaelMallett/drupal_wavesurfer

(Changes by Baker and Rycroft to this module are available in the Scratchpads repository.). The Drupal/Scratchpads code requires a webserver running Apache, PHP and MySQL. Analysis will only be possible if your webserver is allowed access to the BioVeL portal, controlled by the BioVeL project.

### Data sharing

In order to maximise the potential use and reuse of recordings held by BioAcoustica, recordings and metadata are (where licences allow) shared with the EoL (20) through the DarwinCore Archive export facility of Scratchpads (21). DarwinCore Archives are a widely used format for exchanging machine and human readable biodiversity datasets based on the Darwin Core standard (12).

Metadata and files where the copyright belongs to the Natural History Museum, London are made available through the NHM Data Portal (http://data.nhm.ac.uk/dataset/bioacoustica) through the same DarwinCore Archive.

The DarwinCore Archive format also allows the sharing of occurrence records with GBIF and DarwinCore Archive compatible mapping services such as CartoDB (http://cartodb.com).

### Using and contributing to BioAcoustica

You do not need to register to view content on BioAcoustica. To create annotations and run analyses on the platform you will need to register for an account at http://bio.acousti.ca/user. No special permissions are needed for these tasks. If you wish to contribute to BioAcoustica a site administrator will need to grant you permission to do so, instructions on this process, as well as general instructions for using the service, can be found at http://bio.acousti.ca/help.
